# A review of human factors and infusion pumps: lessons for procurement

**DOI:** 10.3389/fdgth.2025.1425409

**Published:** 2025-02-04

**Authors:** Laura Herrero, Marina Cano, Raj Ratwani, Laura Sánchez, Blanca Sánchez, Ramón Sancibrián, Galo Peralta

**Affiliations:** ^1^ Innovation Support Unit, Instituto de Investigación Marqués de Valdecilla (IDIVAL), Santander, Spain; ^2^ MedStar Health National Center for Human Factors in Healthcare, Georgetown University School of Medicine, Washington, DC, United States; ^3^ Clinical Pharmacology Service, Hospital Universitario Marqués de Valdecilla (IDIVAL), Santander, Spain; ^4^ Department of Structural and Mechanical Engineering, Universidad de Cantabria, Santander, Spain

**Keywords:** infusion pumps, patient safety, decision making, organizational, purchasing, hospitals, ergonomics

## Abstract

Integrating advanced technologies like medical devices in healthcare is crucial for addressing critical challenges, but patient safety must remain the top priority. In modern clinical settings, medical devices, such as infusion devices used to administer fluids and drugs, carry risks from use errors, requiring a focus on usability and human factors engineering (HFE). Despite the significance of integrating HFE into technology selection processes, it is often overlooked. A review of five key articles demonstrates how applying HFE principles in procurement strategies can enhance device usability and patient safety. Although designed to reduce medication errors, infusion devices can still cause over-infusion or delays, indicating the need for improved safety features that must be considered in the context of sociotechnical systems. The reviewed studies suggest incorporating HFE in design, purchasing, and implementation to address these issues. The studies highlight various HFE methodologies, showing a wide variation in design, deployment, interpretation, and reporting. This comprehensive examination underscores the importance of standardised evaluations to ensure safer and more effective medical devices, emphasizing the essential role of HFE in advancing patient safety within healthcare settings.

## 1 Introduction

Public procurement in Europe currently accounts for around 14% of gross domestic product. Functioning public procurement markets are crucial for achieving national strategic objectives as they enable the implementation of government policies and improve the competitiveness of quality service strategies (1). It has been recognised that new methods are needed to ensure that public procurement addresses social challenges (2, 3). Patient safety is a crucial challenge in modern healthcare systems that should be considered more in the current formal procurement process (4, 5).

Smart infusion pumps are devices widely used in clinical settings to infuse fluids and drugs in controlled amounts. Different infusion pumps are used for intravenous infusions, in clinical settings, such as volumetric and syringe pumps used for general purposes, patient-controlled analgesia pumps, and insulin infusion pumps. These devices are considered smart when incorporate safety systems that provide high control, accuracy, and precision in drug delivery, with the potential to reduce medication errors and improve patient care, including programmable settings, drug libraries, dose error reduction systems and alarms (6). At the same time, safety issues such as over- or under-infusion and missed or delayed intravenous therapy have been associated with these devices (7). This is why several strategies have been proposed to mitigate infusion pump safety issues by leveraging human factors and ergonomics (HFE) principles during their design, purchasing, and implementation of these technologies (7, 8).

## 2 Human factor and ergonomics and patient safety

According to the International Ergonomics Association, Ergonomics (or human factors) “is the scientific discipline concerned with understanding the interactions between humans and other elements of a system and the profession that applies theory, principles, data, and methods to design to optimise human well-being and overall system performance” (9). Its purpose is to understand how people interact with technology and to study how user interface design affects their interactions with technology (10).

Although some authors distinguish usability from ergonomics and human factors engineering (HFE), these terms have come to be used synonymously (11). The HFE framework considers a systems approach where the user is at the heart of the healthcare system's interactive components such as technology, processes, policies, and people (12). A systems approach considers people to be fallible, unlike the person approach, where people are often blamed for their errors and then trained to prevent them from happening again. In the HFE framework, errors are expected, and the organisation takes steps to understand system components that give rise to errors and address these latent failures that often lead to human error (13).

The HFE discipline began to be applied to U.S. military and space systems. Since then, it has continued to evolve throughout numerous complex systems of all types (12). The term HFE implies the study of worker/machine interrelationships and considering the environment, staffing, and training. The literature describes four primary areas of application of HFE in healthcare technology: design of new technology, improvement of existing technology, evaluation of a technology, and error investigation (14, 15).

HFE seeks to improve clinical performance by understanding the effects of teamwork, tasks, devices, workspace, culture and organisation on human behaviour and skills and the application of this knowledge in clinical settings (9). In this sense, human error is considered to be a cognitive ergonomic element of very high interest for healthcare and considers that the approach to its mitigation should be made from the various perspectives involving HFE with a systemic view (10).

HFE is now recognised as a critical discipline for reducing and mitigating errors (16–18). HFE assumes a limited ability to change the human condition and aims to improve people's working conditions to ameliorate errors (19). HFE helps to understand how healthcare elements interact together, detecting when they fail and affect patient safety. The evidence that the application of HFE techniques has a positive impact on the design and implementation of health technology is diverse and involves a variety of beneficiaries (20).

## 3 HFE and infusion pumps

The Association for the Advancement of Medical Instrumentation and the Food and Drug Administration (FDA) (11) held the Infusion Device Summit in 2010, highlighting 56,000 reported incidents related to intravenous infusions, resulting in 710 deaths, including 14 with serious health effects or death as an effect directly related to the use of infusion pumps. In this conference, it was emphasised that a high percentage of sentinel/drug-adverse events are due to pump use errors and that it is necessary to improve the safety features of the design and define the used protocol to facilitate the users' performance of their tasks properly.

More recently, other studies have confirmed these data. One showed that using infusion pumps leads to frequent errors, reaching 60% of the infusions performed (12). The main errors included software and hardware-related aspects such as incomplete labelling, infusion system not correctly marked, use of unauthorised medication, unused drug library, infusion rate error, omission of intravenous fluids/medications, use of expired medication, and incorrect dosage. Another study in Canada showed that 7.5% of patients admitted to acute hospitals in Canada experienced at least one adverse event, with drug or fluid-related events being the most common type of adverse event, along with those related to surgical procedures (13). Other studies have shown similar frequencies and types of errors. However, for most of them, the adverse events detected with infusion pumps were not serious, and many were due to programming errors such as incorrect drug input, incorrect rate, or incorrect concentration (14–16).

Differences in the safety of different infusion pumps have been detected. In the follow-up of more than 30,000 general-purpose infusion pumps in use (excluding patient-controlled analgesic and syringe pumps) manufactured by 4 different companies, in the network of United States Veteran Affairs centres aggregating more than 150 hospitals, one of the used pumps had five times more use errors than the other three evaluated with substantial added costs of investigating these errors (17). Specific aspects related to infusion pumps impact its safety, such as their instructions for use (18), place of use (i.e., home setting) (19), the type of infusion pumps (i.e., insulin pumps or hepatic artery infusion pumps) (20).

The FDA (7) classifies adverse events related to infusion pumps according to their cause: software problems, alarm errors, inadequate user interface design (“human factors” problems), broken components, battery failure, fire, sparking, charring, or shock. The use of infusion pumps includes several critical actions for patient safety, such as organising infusion tubing, clamping and releasing, and setting flow rates and alarms that can potentially cause severe problems for the patient (21).

Infusion pumps are designed to improve patient safety, although published data about their effects on improving patient safety are conflicting (22). According to a recent retrospective study performed on about 1.5 million infusions in England, it is estimated that when infusion pumps are active, they prevent errors in almost 1 case per 1000 admissions (23). One design element that attempts to improve the safety of these devices is medication error reduction software for smart pumps. The benefits of medication error reduction software include preventing errors such as incorrect rate, incorrect dose, and pump set-up errors, reducing adverse drug effects due to the infusion rate, and enhancing cost-effectiveness (24).

Despite the benefits of medication error reduction software, such systems are often not used in clinical practice, and some studies reveal that the usage rate is between 45% and 80% (23). Smart infusion pump technology itself does not detect drug selection or programming errors, and some errors are derived from the medication error reduction software due to delays in updating the libraries (25) or the wrong selection of medication libraries (26).

In response to the potential impact of adverse events related to infusion pumps, the FDA has developed an infusion pump improvement initiative to establish additional requirements for infusion pump manufacturers to proactively facilitate device improvements and increase awareness among pump users (7), and several recommendations to achieve safer use of infusion pumps have been published (7, 27, 28). These are primarily based on the results of studies that have developed approaches to decrease infusion pump-related adverse events (29, 30) such as the use of medication protocols (31), the application of lean methodology (32), modifications in workflows (33), the intervention of a clinical pharmacist with annotations (34), specific formal training of personnel before pump use (35, 36), strategies to reduce fatigue caused by pump alarms (37) review of libraries by multidisciplinary teams focused on patients treated by oncohematologists (38), inclusion of usability with user participation in the design and testing phase of prototypes (39), and the use of machine learning algorithms to detect alert patterns (40).

## 4 Sociotechnical systems and infusion pumps

Patient safety within healthcare systems cannot be fully understood without considering the role of sociotechnical systems (41). Models like SEIPS (Systems Engineering Initiative for Patient Safety) illustrate how interactions between people, tools and technologies, tasks, and the different environments influence outcomes (10). It highlights that safety is a property of the entire system, not just individual components or actions. SEIPS broader perspective emphasizes that human error alone is never a sufficient explanation for adverse events in healthcare. Instead, it is essential to consider the system's design and the interactions within it (42).

By focusing on how infusion pumps are used in real-world settings, HFE evaluations help identify potential safety issues that arise from these interactions. However, it is equally important to consider other elements of the SEIPS model, such as the tasks being performed, the environmental context, and the organizational policies and procedures that influence how infusion pumps are used (43). These interactions can better anticipate the challenges and opportunities when introducing infusion pumps into clinical settings. Factors such as user interface design, workflow integration, and user training are pivotal in shaping this interaction, highlighting the importance of a holistic approach to technology implementation.

Accordingly, the SEIPS model, the safety of infusion pumps conditioned by the healthcare providers' interaction (usability issues that could lead to errors), the specific tasks, the characteristics of the infusion pumps (design, functionality, and integration), the physical and organizational environment in which infusion pumps are used and the organizational policies and procedures that govern the use of infusion pumps are evaluated (44). This includes understanding the cognitive load on users, their workflow, and the training they receive. For instance, a complex infusion pump interface could lead to programming errors, especially under high-stress conditions (45).

For example, low resourcing or improper training environments may add up to the usability challenges that are brought about while conflicting organization policies for a user create ambiguity as to how the pumps are to be operated or maintained. Furthermore, sociotechnical systems emphasize the feedback loop between users and technology: errors or inefficiencies in one area (e.g., pump malfunctions) often stem from upstream systemic issues, such as procurement decisions, device standardization, or inadequate support infrastructures.

## 5 HFE as a support tool for technology selection

Standard technology selection processes usually do not consider the user and their interactions with the technology, i.e., HFE, within the selection and implementation criteria. In contrast, they focus on thoroughly evaluating the device's utility (i.e., whether it can perform the required functions) and financial viability, i.e., pricing options and competitive tenders (46).

Several organisations have proposed including HFE principles in the design, premarket evaluation, and procurement of health technology to improve its safety. For example, the Western Canada Human Factors Collaborative Guideline (47) proposes to consider an HFE assessment in the procurement of specific medical devices, equipment, and technologies that have implications on patient safety derived from their correct use. In the United Kingdom, the Medicines and Healthcare Products Regulatory Agency's Guidance on the application of human factors and usability engineering to medical devices, including drug-device combination products in Great Britain (8), encourages the inclusion of HFE principles by those involved in risk management, quality and procurement involving medical products.

Despite several recommendations, the application of HFE in selecting health technology has yet to be generalised. Published data from a semi-structured interview in England demonstrated a limited specific evaluation of HFE for selecting health technology (5). Some of the relatively few published cases in which a usability evaluation is developed within a healthcare technology competitive procurement process include the following: the development of a formal usability evaluation in support of purchasing decisions for infusion pumps (48–53), purchase of “point of care” carts (54), of electrosurgical devices (55) of health care catering trolleys (56), an information system for anesthesia (57) all in Canada. Other usability evaluation and procurement studies have included radiotherapy equipment (58), a computer platform for health management with patient participation (59), research-specific information systems, or general institutional information systems not specified in Finland (60). In electronic medical records, a recognised source of medical errors related to usability (61, 62), a recent experience has been reported in France in which a usability study has been developed within a tender (63).

## 6 HFE in infusion pump purchasing decisions

Different studies emphasise the importance of incorporating HFE into infusion pumps to support purchasing decisions as they are health technology with high safety implications derived from their usability (46, 48–53, 64–66). The Western Canada Human Factors Collaborative Guideline (47) and the Medicines and Healthcare Products Regulatory Agency (8) guidelines encourage the inclusion of HFE principles in the procurement involving infusion pumps.

The *Health Safety Investigation Branch*, an organisation within the English NHS that promotes patient safety, has published a report focusing on the acquisition and usability of smart infusion pumps. This report focused on the procurement and usability of *smart infusion pumps, highlighting* questions such as “Should the technology be implemented in this setting?” and “How should the technology be implemented?”. It should be part of the tendering process, as smart infusion pumps are an “off-the-shelf” medical device (rather than a bespoke one), but should not be considered a standardised solution. In other words, the technology cannot be integrated into existing medication practice without significant changes in how medications are prescribed and administered. When selecting smart pump devices, it is essential to consider how this will likely impact practice, although this rarely drives the procurement process (67).

However, HFE methodology is infrequently incorporated in infusion pump procurements. A recent comprehensive review of the public procurement of infusion pumps in Spain for purchasing more than 12,000 infusion pumps, based on an official national public database, found that usability issues are considered relevant for the definition of requirements and selection criteria. Human factors experts and specific methods for evaluating technology were missing, such as field studies, usability walkthroughs, heuristic evaluations, or usability testing (68).

We have reviewed the literature for cases that describe infusion pump procurements involving HFE evaluation to support the selection process. We searched Google Scholar with the following boolean search query: [“infusion devices” AND (“usability” OR “human factors” OR “ergonomics” OR “heuristics”) AND (“procurement” OR “purchasing” OR “medical device acquisition” OR “hospital supply chain” OR “selection”)]. The abstracts of all the retrieved articles in the English language were reviewed, and those that describe an HFE evaluation as support for the procurement of infusion devices were selected, and the original articles were reviewed. The references of the selected articles that mention a selection process of infusion pumps were also analysed.

The search retrieved 233 articles, and only five studies described an HFE analysis of infusion pumps supporting selection. These studies were published in the period 2005–2019 and were conducted in Canada (*n* = 3), the United States (*n* = 1), and Hong Kong (*n* = 1) (48–53) (Table 1). The five studies differ in the type of pumps tendered: standard volumetric and smart pumps (*n* = 2), analgesia pumps (*n* = 2), and epidural pumps (*n* = 1) and the number of different brands evaluated (from 2–4). Detailed information about the procurement (i.e., budget, timing, evaluation experts' profile, and technical pump requirements) is lacking in all five publications, and only one (53) indicates the number of pumps being acquired with the procurement process.

**Table 1:** Published studies using HFE evaluations to support purchasing decisions within infusion pump bidding processes.

Ref	Center	Number/Types of pumps	Methodology	Heuristic test	Usability test	Analysis	Results
(48)	Trillium Hospital, Ontario, Canada	Acquisition of 500 pumps/3 marks	Demonstration by the manufacturer to staff and managers	By one HFE	17 users in total	Heuristic: - number of criteria Usability: - no. errors - no. of critical errors - number of critical errors not detected	The three pumps analyzed A, B and C have in the heuristic test as well as in the usability test
		General purpose smart pumps (A, B, C)	HFE expert evaluation HFE - Heuristic - Task analysis - Usability test (task analysis)	Criteria: 33 criteria in total (including Zhangs’, Nielsen’s and Schneiderman’s modified criteria).	14 nurses		
					- 4 Oncology - 4 Medico-chirurgical - 3 Pediatrics - 3 UCI		Regarding usability A > B > C
					3 Anesthesiologists		
					Location: clinical areas of the hospital		
					Duration: 40 min		
					Pre-training 10 min		
					6 generic tasks		
(49)	University Health Network, Toronto, Canada	3 + 1 brands of patient-controlled analgesia pumps	Task Force creation	By HFE group	Set 1 (3 pumps)	Number of questions to complete the task.	It does not explain the differences between pumps. One of the pumps with built-in omni-directional bar code scanner and better alarm systems superior to the other 3.
			HFE evaluation 2 phases	Zhang’s criteria	- 10 nurses - Pre-training 5 min - 10 tasks each		
			- Heuristic - Usability (task analysis		Set 2: (1 pump):	No. of errors during the task	
					36 nurses		
					45 min each nurse	Task completed without problems	
					Location: clinical areas of the hospital	Answers required to complete the task	
					5 specific tasks based on a clinical case	Significant problems in completing the task.	
(50)	Beaumont Hospitals, Michigan, USA	2 brands epidural pump	Usability test		50 nurses	Categorization of each clinician in the team from 1–5 of: Ease of programming infusion; Volume, identification, troubleshooting and frequency of alarms; Readability of the screen; On-screen instructions.	One of the pumps above, no further details are given.
					Location: high-fidelity simulated environment		
					Total duration 6 h.		
					Support from EHF engineers		
					10 generic tasks.		
(51)	University Health Network, Canada	3 brands of patient-controlled analgesia pumps	Three phases	By one HFE	5–7 participants for each use.	Identification of usability barriers	Detailed description of the heuristic principles violated, recommendations, usage errors and impact on the safety of the 3 pumps. One of the pumps was superior and the test was the criterion that conditioned the selection.
			- Observation - Heuristic - Usability (task analysis)	Zhang’s criteria	Location: high fidelity environment.		
(52)	Hospital Authority, Hong Kong, China	4 brands of general purpose infusion pumps, two of them smart.	Two phases	By 4 experts in HFE, 28 criteria based on Nielsen and Ginsburg criteria	60 participating nurses:	- Task completion time - Frequency of deviations - Frequency of requests for assistance - Users’ perceptions with questionnaire scale 1–6	Detailed comparison of violations, their severity, task time, frequency of need for assistance, perceptions of use.
			- Heuristic - Usability (task analysis) with		-12 operating room -30 Medical Dept. -UCI: 12 -Pediatrics: 6		
				Questionnaire.	Nurses selected by availability and previous experience		It refers that the results were useful for selection
				Duration ∼75 min	Stratified by unit and experience (=<8 vs. >8 years).		
					7 very specific tasks (with subtasks) described based on a clinical case		
					Location: high fidelity environment.		
					75 min each nurse		

The methodology used for the HFE analysis in the five studies was diverse. A heuristic test was developed in four studies, and usability testing was in five. A heuristic test was deployed using 10–33 criteria. Zhang's criteria were used in four cases combined with others, as Nielsen's and Schneiderman's adapted criteria in two cases. Custom criteria adapted from the standards of regulatory bodies were also applied in two. The number of HFE experts who supported the heuristics ranged from 1–4.

Usability testing was developed in all five cases. In these studies, usability testing was developed in hospital wards (*n* = 2) or high-fidelity scenarios (*n* = 3), and HFE experts supported it in all cases. A brief usability testing pretraining was performed in two cases (5–10 min). In the usability test, there were relevant differences among the studies in the groups of testers, composed of different profiles of clinical professionals. Nurses were present in the four studies that specified group user profiles; only one included anesthesiologists, and none included other medical profiles. Specific clinical areas or specialties referred to as identifiers for group users for nurses were applied in two studies that included oncology, intensive care, surgical areas, medical departments, and paediatrics. Stratification by previous clinical experience of users was used in one.

Tasks selected for usability tests are reflected in Table 2. The number of tasks in usability testing in the four studies that identified this issue ranged from 5–10. The complexity of the tasks was quite variable. It included some specific and simple tasks (i.e., “turn device on”, “attach bolus cord”) or another complex (“set an infusion for a loading dose of digoxin 0.25 mg in 100 ml NS over 30 min or 1, 2, or 4 h in pump B”) that implied several subtasks. In two usability tests, tasks were based on a clinical case.

**Table 2:** Usability tasks applied in the five procurements studies analyzed.^a^

Tasks	References of the studies that apply the task
Examine device interface and point out various buttons and functions	(50)
Turning on and off the pump	(48)
Loading and unloading a set	(48, 50)
Programming a basic infusion	(48, 50)
Programming a drug infusion from scratch	(48, 52)
Programming a drug infusion from a drug library	(48, 52)
Programming an infusion on a secondary line	(48, 52)
Clear your programming	(49)
Check your settings (include a misprogrammed)	(49, 50)
Switch the medication	(49, 52)
Start infusion	(50)
Give bolus	(50)
Pause infusion	(50, 52)
Increase dose	(49, 52)
Decrease dose	(49)

^a^ Some of the tasks are described in the original studied as a complex ones based on clinical cases (i.e.: “adjust the remaining Fucidin solution of 230 ml to be infused over 4, 6, 7, or 8 h because the patient complained of mild itchiness over arms”).

In the analysis of the HFE studies, the methodology was also quite variable. The results of the four heuristic studies were based on the number of heuristic criteria violated, and the numeric results of this test were presented only in two. Two cases also presented a list of usability strengths and weaknesses of infusion pumps detected in heuristics. Usability test results analysis was based mainly on the number of errors (*n* = 2), number of critical errors (*n* = 1), number of questions to complete a task (*n* = 3), number of tasks completed with problems (*n* = 1), problems to complete a task (*n* = 1), subjective classification of easiness in specific issues (*n* = 2), task completion time (*n* = 1), frequency of deviations (*n* = 1).

The time requirements for the studies are considered in the reports, but with different approaches, making comparisons difficult. For example, Lau (50) indicates that the overall usability test time for 50 nurses was 6 h, while Ginsburg (48), Ladak (49), and Liu (52) indicated that an individual session lasted around 40, 45, and 75 min, respectively. Independently of the time for HFE evaluation, the overall duration of the procurement may be much larger, as described in Landak (49), which indicates that the process was time-intensive and lasted over three years.

## 7 Discussion

Current research underscores the value of HFE in assessing health technologies, particularly for user-centric patient safety concerns, as seen in infusion pumps. These evaluations are pivotal in guiding bidding decisions. However, the literature reveals that the application of HFE in procurement processes, though beneficial in identifying optimal technology, understanding risk factors and training needs, and implementing improved strategies for patient safety, still needs to be more consistently applied and developed (5, 68, 69).

Health technologies evaluation must be considered from the perspective of sociotechnical systems theory, particularly the SEIPS model (Systems Engineering Initiative for Patient Safety). SEIPS considers how interactions between people, tools and technologies, tasks, and the environment determine how processes can yield both safe and unsafe outcomes. The SEIPS model is anchored in HFE and provides a comprehensive framework for examining how different components of healthcare systems interact to influence patient safety (10). Within this model, HFE evaluations of infusion pumps tackle one critical element: the interaction between users and technology. By focusing on how infusion pumps are used in real-world settings, HFE evaluations help identify potential safety issues that arise from these interactions (41, 45). However, it is equally important to consider other elements of the SEIPS model, such as the tasks being performed, the environmental context, and the organizational policies and procedures that influence how infusion pumps are used (43).

The broader use of the SEIPS model envisions transforming reactive safety management into proactive safety management. Focusing on the health system as a whole would allow for the development of processes and technology that are not just safe but also adaptive under changing conditions and stressors. Such a systems-oriented approach helps alleviate risks, cut down the occurrence of errors, and support well-responsive as well as reliable delivery of care by health professionals (44). Integrating these perspectives into HFE and procurement processes can lead to more effective and sustainable safety improvements. By focusing on the system as a whole and understanding its interactions, we can design more resilient systems that support safe care delivery and reduce the occurrence of errors (70).

A regulatory framework encouraging comprehensive HFE assessments can lead to more consistent and thorough evaluations of high-risk devices like infusion pumps. This consistency promotes safety, as comprehensive HFE assessments can identify and mitigate risks, reducing medication errors, over-infusion, or therapy delays. By focusing on user interaction, standardised HFE assessments improve usability, identify potential risks, and recommend improvements to enhance safety, making medical devices easier to operate and less prone to errors (71).

Implementing regulatory frameworks in healthcare technology requires a multi-step process that involves close collaboration among healthcare providers, regulatory agencies, and medical device manufacturers. Establishing clear HFE standards for high-risk devices, such as infusion pumps, is crucial to ensuring a consistent approach to assessment and evaluation (47). A standardised approach to HFE can lead to more reliable and user-friendly medical devices, reducing the likelihood of use errors and adverse events.

Additionally, the complexity of HFE assessments necessitates the training of multidisciplinary teams. Medical devices, especially those like infusion pumps, involve a complex interplay of technology, human interaction, and healthcare practices. A multidisciplinary team can navigate this complexity, combining the insights necessary to evaluate usability, identify risks, and recommend design improvements (9). These teams should comprise professionals with expertise in healthcare, engineering, ergonomics, and user experience design. Their collective skills are crucial for thorough HFE testing and implementation, a resource-intensive process demanding meticulous planning and prioritization.

The reviewed data in the published HFE evaluations supporting the procurement of infusion pumps indicate a wide variation of HFE design, deployment, interpretation, and reporting study methodologies. These differences may be provoked partly by local resources, activities, and needs differences. Also, differences in the characteristics of the evaluated equipment may contribute to them. These disparities make the studies' interpretation, comparison, and reproducibility complex.

Implementing these proposals is time-consuming and implies an adequate team, including HFE experts, and planning for accomplishing times and the quality of the evaluation. Also, it could significantly elevate patient safety standards. The diverse skill sets of a multidisciplinary team allow for a more holistic view of the challenges and potential solutions in medical device assessment. This comprehensive approach leads to more robust and reliable results, ensuring that devices meet safety and usability standards. By ensuring rigorous, standardised HFE evaluations in procuring critical devices like infusion pumps, patient safety can be substantially improved, reducing the risk of device-related errors and enhancing overall healthcare outcomes.

Sociotechnical models such as SEIPS stress the need for understanding the entire “big picture”—the environment, organizational policies, and user interactions—to spot and circumvent risks. This double emphasis upon not just “fit for purpose” device usability but also wider factors that bear upon device use may create a safer system; a resilient system that might well proactively cut down errors and then mold itself around dynamic clinical settings (72). The impact of improved usability on infusion pumps and the sociotechnical approach to their implementation is enormous. The human-computer interface embedded inside these devices would lessen the cognitive burden on medical practitioners, minimize programming errors, and support quicker and more accurate job accomplishment. For example, infusion devices supported with good user interfaces and error prevention applications may cut the critical error rates related to the therapies they support by as much as fifty percent, thereby having such a direct impact on patient safety (48).

By integrating the evaluation of infusion pumps into the SEIPS model, we can ensure that all aspects of the system are considered. This comprehensive approach helps identify not only the devices' usability issues but also how they fit into the broader healthcare system. It allows for a more holistic understanding of the factors that contribute to both safe and unsafe outcomes, ensuring that interventions address the root causes of errors.
